# Understanding silence in adolescence and young adulthood: a mixed methods systematic review on selective mutism

**DOI:** 10.1007/s00787-025-02951-y

**Published:** 2026-02-20

**Authors:** Rachel de Jong, Anja van der Voort, Wessel Aben, Maretha V. de Jonge

**Affiliations:** 1 Department of Clinical Neurodevelopmental Sciences, Leiden University, Leiden, the Netherlands; 2 Youz Specialist Care for Youth and Families, Den Haag, the Netherlands

**Keywords:** Selective mutism, Adolescents, Young adults

## Abstract

The aim of this study was to identify the characteristics of adolescents and young adults with selective mutism (SM) and their specific (treatment) needs, as well as those of their environment. We conducted a mixed methods systematic review of scientific databases APA PsycINFO and MEDLINE until February 2025. Descriptive studies were included if they incorporated information on individuals between 10–24 years old with a current diagnosis of SM. Two independent reviewers extracted data using combined deductive and inductive coding, with categories finalized by consensus. Data were synthesized using a convergent segregated approach. A total of 749 records were identified, of which 40 studies (*n* = 54 participants) met inclusion criteria. Methodological quality varied, with 27.5% studies rated as high quality, 65% as moderate, and 7.5% as low. Key findings showed that SM manifests differently in adolescents and young adults compared to children, and therefore requires a different approach to treatment. The reviewed literature shows that adolescents and young adults with SM have a later onset, longer diagnostic delays, and a prolonged history of negative reinforcement of symptoms from accommodations by others. Consequently, SM tends to be more therapy-resistant, requiring greater patience and diligence from therapists.

## Highlights

SM in older youth has a later onset and longer diagnostic delays.

SM in older youth is more therapy-resistant due to prolonged negative reinforcement.

There is a need for age-appropriate approaches in both research and treatment of SM.

## Introduction

Youth with selective mutism (SM) suffer from a psychiatric condition that is characterized by an absence of speech in specific public situations in which they are expected to speak (e.g., at school), while in other situations (e.g., at home), their production of speech is normal. This ‘non-speaking behavior’ probably serves as an emotion regulation strategy. By remaining silent, the individual reduces anxiety or other negative emotions in stressful or otherwise challenging situations (e.g., school or work), and is thus able to decrease emotional and physiological distress [57]. The position of SM has been altered in the last edition of the handbook for the classification of mental disorders, the Diagnostic and Statistical Manual of Mental Disorders (DSM-5, [5]). It was removed from ‘Disorders of childhood and adolescence’ and placed under ‘Anxiety disorders’. This led to two important changes in the interpretation of symptoms of SM. First, it highlights the anxious etiology of the disorder and second, it allows more room for diagnosing SM in (young) adults.

While SM typically begins in early childhood, it sometimes persists into adolescence or young adulthood. The mean age of onset of SM varies between 2 and 4 years [72]. However, the mean duration of SM is found to be 9 years with a range of 3 to 15 years [53], which indicates that some children with SM will still suffer from the disorder as an adolescent or young adult. Correspondingly, in interviews with a small group of recovered individuals (ages 31–60), SM persisted into adolescence or even young adulthood in 5 of the 6 adults [48]. This is also reflected in prevalence numbers, as an estimated 7.1–18.9 per 1000 children (0.7–1.9%) aged 5 to 8 years are diagnosed with SM [9, 39], while an estimated 1.8 per 1000 older children (0.18%) aged 8 to 15 years are (still) diagnosed with SM [33, 59]. Cases of SM in adolescents or adults over 15 are rare, possibly because older individuals can more easily avoid social situations where they would be required to speak [38]. As a result, research on SM in adolescents and young adults is scarce, limiting our understanding of its presentation and course in these older age groups.

What is known, is that even in cases where SM is apparently cured, individuals often continue to struggle with symptoms of shyness, speech inhibition, and/or social anxiety disorder into adolescence and adulthood [46]. This is illustrated by two studies [53, 63] that retrospectively examined groups of respectively 41 and 33 young adults (mean age 20–21 years) who were diagnosed with SM as children (mean age 8–9 years). Only in 39–58% of the cases full remission was reported. The other 42–61% improved slightly or markedly but still struggled with communication in social situations. Mutism within the core family (i.e., the individual does not speak with family members) at time of referral was a predictor of poor treatment outcome at follow-up [53], as were immigrant status and bilingualism [63]. Many young adults still described themselves as less independent, less motivated for school or work, less self-confident, and less mature and healthy in comparison to matched controls with other emotional disorders [53]. A more recent follow-up study that included somewhat older young adults (mean age 26–28 years), showed that those who experienced SM and did not feel cured (*n* = 41), had higher interpersonal anxiety, lower communication skills, and more difficulty with speaking to others than those who experienced SM and did feel cured (*n* = 36) [68]. However, even the latter group still showed higher interpersonal anxiety, lower communication skills and less self-esteem than adults who never experienced SM. Tomohisa and colleagues [68] found that the feeling of being cured of SM was affected by the level of interpersonal anxiety and whether adults still felt difficulty in speaking to others. In addition, a recent review concluded that older age at first diagnosis and parental psychopathology might predict greater impairment later in life [34]. Together, these studies stress the need for a better understanding of SM symptoms and its treatment in these older age groups specifically.

In the few quantitative studies that included both younger and older individuals to get an understanding of SM across the life span, it was concluded that older children (10 + years) were overrepresented among poor treatment responders [23, 46], and were more often treated as inpatients [53] compared to younger children (10- years). Also, adolescents experienced a significantly longer lag time than pre-school or school-aged children (7.5–24 months [13, 27],), with on average 30 months between the onset of SM and receiving the diagnosis [27], indicating that SM may have been overlooked in these older individuals. This is worrisome, given that compared to adolescents with other internalizing disorders, adolescents with SM revealed higher levels of internalizing problems like agoraphobia, and considered themselves as significantly more withdrawn and having more social problems [28]. In line with this, a recent survey study found that adolescents with SM not only used verbal communication less frequently than those without SM, but also engaged less in written and computer-mediated communication, such as messaging on social media platforms [20]. In sum, SM in adolescents appears to manifest differently from SM in younger children and from other internalizing disorders in adolescents. It might therefore require a different approach to its assessment and treatment in these older age groups particularly.

So far, however, no quantitative cohort studies have exclusively focused on adolescents or young adults with SM. Fortunately, descriptive studies including adolescents or young adults with SM have been published since the late 1960s. Yet, a systematic overview of the findings of these descriptive studies is lacking, making it difficult to draw firm conclusions on whether the findings observed in children with SM can be generalized to older individuals with SM. Therefore, the current study set out to offer a comprehensive initial overview of the descriptive literature on adolescents and young adults with SM. Because quantitative studies represent symptoms in a generalized form that may not fully capture participants’ actual experiences, there is a need to focus on other types of research that can provide contextual insights from the adolescents and young adults themselves, as well as from important people in their environment. As individuals with SM are sometimes unable to communicate their needs and desires, gathering information from significant others could potentially uncover more important aspects of SM.

The aim of this study was to identify the characteristics of adolescents and young adults with SM and the specific (treatment) needs of themselves as well as of their environment, to provide a better understanding of SM in this so far overlooked group of individuals. To reach this goal, we systematically reviewed the descriptives of these individuals, their treatment and their own and others’ perspectives on their condition and its treatment, as reported in descriptive studies on SM among adolescents and young adults published since the late 1960s. Exploratively, this study set out to identify implications for the assessment, treatment, and future research into ‘non-speaking behavior’ in older individuals. With this aim in mind, the following research questions were formulated:

1. What characteristics of adolescents and young adults with SM have been documented by researchers, therapists, people in their environment, and the individuals themselves?
2. What types of treatments and their effects for adolescents and young adults with SM have been described by researchers, therapists, people in their environment, and the individuals themselves?
3. What perspectives on and experiences of adolescents and young adults with SM have been reported regarding their condition and its treatment?

## Methods

A mixed methods systematic literature review and descriptive analysis was reported in concordance with the Preferred Reporting Items for Systematic reviews and Meta-Analyses (PRISMA; [51]) guidelines, see Appendix. Note that this review was not pre-registered, so no registration information could be provided for this review.

### Search strategy

To decide whether a study was eligible for inclusion in the review, the following criteria were used: 1) The paper’s participant(s) is/are adolescent(s) or young adult(s) (10–24 years old) with a current diagnosis of SM that could not better be explained by a psychotic of neurological disorder or by an intellectual disability (IQ < 70); 2) The paper contains information about (an) individual participant(s) in a quantitative or qualitative design (e.g., a single case experimental design study, case control study, case study, case report, survey, observational or interview study); 3) The paper is written in English or Dutch; 4) The paper is either an academic journal article or dissertation, not a study protocol, instrument validation, review article, response to the editor, book (review) or a book chapter. Unpublished dissertations were included in the search results to reduce publication bias.

The scientific databases APA PsycINFO and MEDLINE were used through EBSCOhost for finding relevant published and grey literature up to early 2025. Keywords used for searching these databases were *elective; mutism, AND, adolescen*; youth; young. As selective mutism was called elective mutism until the DSM-IV, both elective and selective mutism were included as search terms. To be able to find all relevant literature on adolescents and young adults with SM, we included different ways to refer to this age group as search terms. The World Health Organization (WHO) defines 'Adolescents' as individuals in the 10–19 years age group, ‘Young adults’ as the 19–24 year age group, and 'Youth' as the 15–24 year age group, while 'Young People' covers the age range of 10–24 years. These search terms were agreed upon by all authors and were identical for both databases. Search terms had to be present in the abstract for a record to be selected for further review.

### Study selection

**Fig. 1:**
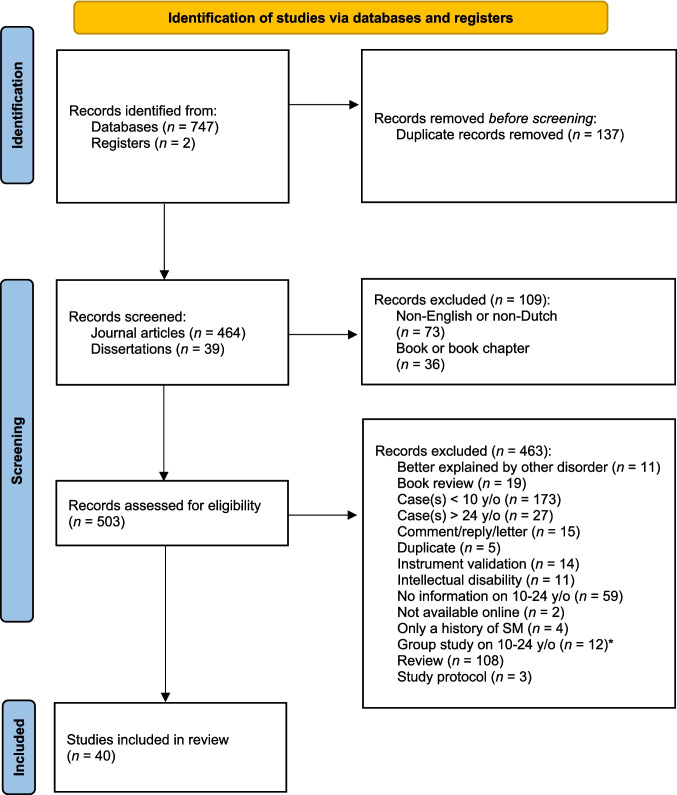
PRISMA 2020 Flow diagram. *Described in introduction

The primary search was conducted in June 2023 and covered the period from 1965 to June 2023 and resulted in a total of 748 records. To ensure comprehensiveness and currency, an additional search using the same eligibility criteria was conducted during the manuscript preparation phase, covering the period from July 2023 to February 2025. One additional study was identified during this period, resulting in a total of 749 records (see Fig. 1). The search results were stored and managed in Microsoft Excel software. Before screening, duplicates were removed, which left 612 records remaining. Thereafter, records in other languages than English or Dutch were deleted, resulting in 539 records remaining, all of which were in English apart from two Dutch publications. Finally, books and book chapters were deleted, which left 503 records remaining, all of which were academic journal articles (464) or dissertations (39) published or placed online between 1965 and 2025.

The first stage of screening was based on an examination of the title and abstract against the eligibility criteria. This first stage of screening resulted in a first selection of 52 studies. The second stage of screening was based on examination of the full text against the same inclusion criteria. This second stage of screening resulted in a final selection of 40 studies (38 academic journal articles and 2 dissertations). The first and second stage of screening were conducted independently by the first author. The last author independently conducted the second stage of screening. Any discrepancy in study selection was resolved through discussions, and all the authors verified the entire selection process, see the flowchart in Fig. 1.

### Data extraction, coding, and synthesis

Key data from each case study were systematically extracted into a standardized data extraction form (see Table 1). This form included (a) study information; (b) participant characteristics; (c) treatments; and (d) perspectives on and experiences of adolescents and young adults with SM. All included papers were then entered into a qualitative data analysis software (Atlas.ti 23) for coding. Codes were developed deductively as well as inductively, allowing predefined codes to be adapted and new codes to be identified based on the data. Coding was conducted per participant, so per included study all data were extracted from all participants who met the inclusion criteria. Information regarding participant characteristics, treatments for, perspectives on, or experiences with SM, were coded only if clearly described by informants, clinicians, or researchers and if they indicated relevant to SM. Across the four dimensions (a) till (d), items were coded into a binary, categorical or numerical scale. All included studies were independently coded by the first author. The third author independently coded half of the included studies. Any discrepancy in codes was resolved through discussions, and all the authors verified the entire coding process by checking whether the wording of the categories were appropriate. Because our research questions could be addressed by both quantitative and qualitative research designs, this study followed a convergent segregated approach to data synthesis [64].

**Table 1:** Overview of included studies and participants

	Year, author	Type of study	Location	Gender	Age	Diagnoses*	Setting	Perspective	Approach	Quality
1	2025, Shimoni et al. [60]	Case report	Israel	Female Female Female	12 10 14	SM, ASD, ADHD SM, ASD SM, ASD	Out-patient clinic	Therapist	Eclectic	5/8^b^
2	2024, Casas and Conn [15]	Case study	USA	Trans Trans	18 14	SM, PTSD SM, PTSD	Out-patient clinic	Therapist	CBT	8/8^b^
3	2023, Keville et al. [35]	Interview study	UK	Male Male Trans Female Female Male Male Female	14 16 15 16 17 18 13 16	SM, SAD SM, ASD, GAD SM, ASD SM, ADHD SM, ASD, SAD SM, ASD SM, ASD SM, ADHD, SAD, GAD	Research setting	Parents	System	8/10^d^
4	2021, Cengher et al. [16]	SCED	USA	Female	11	SM, ASD, ADHD, GAD	Out-patient clinic	Therapist	CBT	8/14^a^
5	2018, Valaparla et al. [70]	Case report	India	Male	11	SM, ASD	In-patient clinic	Therapist	CBT	2/8^b^
6	2018, Holka-Pokorska et al. [30]	Case report	Poland	Male Male	17 14	SM, ASD, MDD SM	In-patient clinic	Therapist	Biological	3/8^b^
7	2017, Lawrence [40]	Case study	UK	Male	12	SM	Education service	Therapist	CBT	8/8^b^
8	2017, Nashman-Smith [45]**	Interview study	USA	Female	13	SM	Education service	Parent; teacher	Eclectic	8/10^d^
9	2016, Eugene et al	Case control study	Poland	Female	14	SM	In-patient clinic	Therapist	Biological	6/10^c^
10	2016, Albrigtsen et al. [3]	Interview study	Norway	Male Male	14 14	SM, SLD SM, SLD	In-patient clinic	Parent, patient	System	8/10^d^
11	2015, Walker and Tobbell [74]	Interview study	UK	Female Male	23 21	SM SM	Research setting	Patient	Eclectic	9/10^d^
12	2015, Serra et al. [58]	Case report	Italy	Female	17	SM, SAD	Out-patient clinic	Therapist	Biological	4/8^b^
13	2014, Fernandez et al. [25]	Case report	Philippines	Male	10	SM	Other: art therapy	Therapist	Other: art	2/8^b^
14*	2013, Berko & 2013, DeMille [10, 21]	Case report	USA	Female	16	SM	Out-patient clinic	Therapist	Psycho-dynamic	5/8^b^
15	2013, Bunnel et al	Case study	USA	Female	17	SM, SAD	Out-patient clinic	Therapist	CBT	8/8^b^
16	2012, Christon et al. [17]	Case study	USA	Female	15	SM, SAD	Out-patient clinic	Therapist	CBT	8/8^b^
17	2011, Shriver et al. [61]	Case study	USA	Male	10	SM	Out-patient clinic	Therapist	CBT	6/8^b^
18	2008, Turkiewicz et al. [69]	Case report	Brazil	Female	17	SM, SP	Out-patient clinic	Therapist	Biological	4/8^b^
19	2008, Beare et al. [7]	SCED	USA	Male	12	SM	Education service	Researcher	CBT	7/14^a^
20*	Omdal and Galloway [49], [50]	Interview study	Norway	Male Female	11 13	SM SM	Research setting	Researcher	Psycho-dynamic	6/10^d^
21	2007, Vecchio and Kearney [71]	Case study	USA	Female	10	SM, SAD, GAD	Out-patient clinic	Therapist	CBT	8/8^b^
22	2007, Park et al. [52]	Case report	Canada	Female	12	SM, SAD	In-patient clinic	Therapist	Biological	6/8^b^
23	2007, Dallos [19]	Case report	UK	Male	15	SM	In-patient clinic	Therapist	System	3/8^b^
24	2006, Fisak et al. [26]	Case study	USA	Male	10	SM, SAD	Out-patient clinic	Therapist	CBT	8/8^b^
25	2005, Boggs [12]**	Case study	USA	Female	10	SM	Research setting	Researcher	Eclectic	8/8^b^
26	2002, Jainer et al. [31]	Case report	UK	Female	24	EM	Out-patient clinic	Therapist	Biological	6/8^b^
27	[67]	Case report	Denmark	Female	17	EM	In-patient clinic	Therapist	Biological	4/8^b^
28	1999, Rye and Ullman [55]	Case study	USA	Male	13	EM	Out-patient clinic	Therapist	CBT	6/8^b^
29	1997, Kristensen [36]	Case report	Norway	Male	12	EM, SLD	Out-patient clinic	Therapist	Biological	4/8^b^
30	1995, Watson [75]	Case study	UK	Female	10	EM	Other: speech/language therapy	Therapist	Other: speech/language	5/8^b^
31	1995, Motavalli [42]	Case report	Turkey	Female	12	EM	Out-patient clinic	Therapist	Biological	5/8^b^
32	1992, Black et al	Case report	USA	Female	12	EM	Out-patient clinic	Therapist	Biological	6/8^b^
33	1986, Albert-Stewart [2]	Case study	USA	Male	13	EM	Out-patient clinic	Therapist	CBT	5/8^b^
34	1985, Wilkins [78]	Case report	UK	Male Male Male	13 14 10	EM EM EM	Out-patient clinic	Therapist	System	2/8^b^
35	1979, Wergeland [76]	Case report	Norway	Female	10	EM, OCD	In-patient clinic	Therapist	Psycho-dynamic	3/8^b^
36	1979, Ambrosino et al	Case report	USA	Female	10	EM	Out-patient clinic	Therapist	Psycho-dynamic	6/8^b^
37	1977, Colligan et al. [18]	Case report	USA	Male	11	EM	Out-patient clinic	Therapist	CBT	5/8^b^
38	1969, Halpern [29]	Case report	USA	Male	11	EM	Other: speech/language therapy	Therapist	Other: speech/language	6/8^b^

* = two different studies including the same participant(s). ** = dissertation. SM = selective mutism; EM = elective mutism (SM was called EM until the DSM-IV); PTSD = post-traumatic stress disorder; ASD = autism spectrum disorder; ADHD = attention deficit hyperactivity disorder; GAD = generalized anxiety disorder; SLD = specific language disorder; SAD = social anxiety disorder; SP = specific phobia; SCED = single case experimental design. ^a^ = RoBiNT Scale for SCEDs (1–4 low; 5–10 medium; 11–14 high); ^b^ = JBI Checklist for Case Reports (1–3 low; 4–6 medium, 7–8 high); ^c^ = JBI Checklist for Case Control Studies (1–3 low; 4–7 medium; 8–10 high); ^d^ = JBI Checklist for Qualitative Research (1–3 low; 4–7 medium; 8–10 high). Approach refers to the theoretical framework used to guide the research

For qualitative data extracted from the included studies, descriptive themes and integrated findings are presented in tables and summarized in the text with references to the relevant study numbers. We applied thematic synthesis [66] to categorize the qualitative data. Themes were generated by grouping similar codes and examining patterns across studies, and were refined iteratively through discussions among the authors. To manage the large number of features reported in the original studies, participant characteristics, treatments, and perspectives were organized into broader clusters through a stepwise process. First, all participant features were extracted verbatim. Features that differed in terminology but reflected similar concepts (e.g., “social skills” vs. “social communication”) were compared for conceptual overlap. When deemed to represent the same underlying clinical phenomenon, these features were integrated into broader clusters. Clustering was conducted by the first author and informed by established diagnostic frameworks, observed data patterns, and relevant literature. Special consideration was given to age-specific aspects, recurring challenges, and the transferability of findings across contexts. To identify both commonalities and differences across the case studies, we performed cross-case comparisons on key variables, such as participant characteristics, treatments and perspectives.

Where studies provided quantitative data (e.g., symptom measures or outcome metrics), these were integrated into the synthesis using a narrative summary approach. When possible, tables were used to demonstrate the frequencies for the total number of participants. This approach was preferred over reporting the total number of studies, as some studies described multiple participants. Quantitative results were presented alongside qualitative insights to provide a comprehensive understanding of the cases. Given the low use and lack of consistency in assessment of symptoms and treatment effects reported among the different participants, no meaningful quantitative meta-analysis comparing the different assessment or treatment outcomes could be conducted.

### Critical appraisal

To ensure the robustness of our synthesis, the quality of included case studies was assessed using the Joanna Briggs Institute (JBI) Critical Appraisal Checklist for a) Case Reports; b) Case Control Studies; c) Qualitative Research (JBI 2020) [6], and d) RoBiNT Scale for SCEDs [65]. The first author independently reviewed all included studies and performed the quality ratings. The third author independently performed quality ratings on half of the included studies. Results were compared and differences of opinion were discussed and resolved by consensus. Quality ratings for each included study can be found in Table 1.

### Data analysis

Data analysis followed a descriptive, integrative approach aimed at systematically mapping the characteristics, treatments and perspectives of adolescents and young adults with SM across diverse study types. Due to the variety of methodological approaches in the studies included and the exploratory character of this review, a descriptive strategy was considered most appropriate to provide a comprehensive overview of the existing evidence.

## Results

### Study information

The included studies (38 published, 2 unpublished) are described in a schematized summary, shown in Table 1. The majority of the 40 included studies are case reports (*n* = 20, 50%) or case studies (*n* = 12, 30%) written from a therapist perspective (*n* = 30, 75%) and a cognitive behavioral theoretical approach (*n* = 13, 32.5%). According to the criteria used, three studies are of low quality (*n* = 3, 7.5%), while the majority is of medium (*n* = 26, 65%) or high quality (*n* = 11, 27.5%).

As shown in Table 1, the included studies contain 54 unique cases aged between 10 and 24 years (25 man (46.3%), 26 woman (48.1%), and 3 transgender (5.6%)), of which a majority of 36 adolescents was aged up to 15 years (66.7%). Twenty-five of the participants were seen in an out-patient setting (46.3%)*,* thirteen in a research setting (24.1%), ten in an in-patient setting (18.5%), three in an education setting (5.6%), and three in another setting (5.6%). As shown in Table 2, SM was present when participants were on average six years old, but only got diagnosed at an average age of nine and a half years, indicating an average lag time of about three and a half years. The mean duration of SM was found to be seven years, and almost half of the participants suffered from a co-occurring condition besides SM (*n* = 24, 47.1%), with autism spectrum disorder (ASD, *n* = 11, 20.4%) and social anxiety disorder (SAD, *n* = 9, 16.7%) being most prevalent (see Table 1 for prevalence).

**Table 2:** Descriptive statistics of age related variables (in years)

	*M (SD)*	*Median*	*Min*	*Max*
Age (*n* = 54)	13.78 (3.28)	13	10	24
Age of onset (*n* = 42)*	6.14 (3.92)	5	2	17
Age of diagnosis (*n* = 32)	9.47 (4.89)	10	2	18
Lag time (*n* = 24)	3.35 (3.52)	2	0^**^	13
Duration of SM (*n* = 42)	7.36 (4.10)	8	0^**^	21

*Age of onset is the age that the first symptoms of selective mutism are reported. **Lag time and duration of SM shorter than one year but longer than one month

For the assessment of SM symptoms, semi-structured interviews were reported to be used in seven participants, including the Anxiety Disorders Interview Schedule for Children for DSM-IV (ADIS-IV-C/P; [1]) [12, 15, 21, 24, 32], the Schedule for Affective Disorders and Schizophrenia for school-age children-present and lifetime version (K-SADS-PL; [32]) [18], and one unspecified interview [4]. The Selective Mutism Questionnaire (SMQ; [8]) was reported to be used in three participants [12, 16, 25], while structured observation of speech behavior was reported to be used in six participants [4, 15, 17, 19, 24, 28].

### Participant characteristics

Participant characteristics are summarized in Table 3. Difficulties or delays in early language production or processing were reported for twelve participants (22.2%). Second, problems in social interaction or with social relations were reported in almost half of the participants (*n* = 25, 46.3%), and often co-occurred with fear of or anxiety about social situations, reported in about one-fifth (*n* = 12, 22.2%) and one-third (*n* = 19, 35.1%) of the participants respectively. In line with this, a third of the participants were reported to avoid social interaction (33.3%). Also, two-fifth of the participants were reported to be shy (*n* = 22, 40.7%), either by showing an inhibited temperament from an early age (*n* = 13, 24.1%), and/or by withdrawing from unfamiliar situations or people (*n* = 14, 25.9%). In addition, for nine participants a negative affect was reported (16.7%). Despite the internalizing pattern described here, a quarter of the participants were reported to show disruptive behavior while growing up (*n* = 13, 24.1%).

**Table 3:** Reported participant characteristics with descriptions, frequencies (% of total participants (*n* = 54)*), examples, and relevant article numbers

Characteristic	Description	Frequency	Prototypical examples	Articles
Pre/perinatal problems	Prenatal risk factors or risk factors in the perinatal period	5.6%	“Mother recalled she endured severe stress during pregnancy” “AJ was born prematurely, with perinatal complications”	1, 25, 30
Somatic complaints	Physical symptoms that a person experiences, which may or may not have a clear medical cause	16.7%	“He had bodily reactions such as stomach and headache” “He had migraine headaches which began when he was 8” “She had difficulty falling asleep”	2, 10, 15, 16, 21, 25, 29, 34
Sensory complaints	Sensitivity to subtle stimuli and/or being easily over aroused by stimuli	16.7%	“Noise has a big impact on him” “D is particular about her attire and food”	1, 3, 5, 6, 22, 25, 34
Motor complaints	Problems with movement from involuntary to goal-directed actions	9.3%	“The neurological examination showed a slight hypotonia” “His gross-motor functioning was poor”	1, 29, 33
Speech/language complaints	Difficulties in the verbal production of language or the processing of communication	22.2%	“N was diagnosed with an articulation disorder” “The acquisition of language was significantly delayed” “She was able to speak 3 to 4 four sentences at 3 years of age”	1, 6, 10, 15, 20, 29, 30, 31, 33, 38
Toilet complaints	Problems with toilet training, constipation, enuresis, and encopresis	7.4%	“She was trained at 4 years but with diurnal enuresis until 7” “Till 9 years of age encopresis was observed”	6, 14, 15, 29
Cognitive complaints	Difficulties in information processing and reasoning skills	9.3%	“Difficulty with making decisions and concentrating” “Rigidity in problem solving and slow processing speed”	1, 2, 10, 25
Social complaints	Problems in social interaction or with social relations	46.3%	“Social communication skills were poor compared to siblings” “The patient always had a very small group of peers and limited relationships” “They told us that they have been bullied” “She had a very restricted scope of social activities”	1, 2, 5, 6, 7, 10, 11, 12, 15, 16, 20, 21, 22, 24, 25, 27, 28, 29, 31, 32
Emotional complaints: fear	Emotional response to a perceived threat or danger	22.2%	“Stressful fear-inducing nature of social interactions” “Fear of being negatively evaluated” “Leaving her paralyzed with fear and vulnerability”	2, 3, 12, 14, 15, 16, 20, 21, 24, 25, 28, 36
Emotional complaints: anxiety	Emotional state characterized by excessive worry or nervousness in anticipation of future events or uncertain outcomes	35.1%	“He reported feeling anxious and on guard most days” “Her worry was primarily in the context of social situations” “Making her anxious and afraid to be with other people” “M was shy, anxious and withdrawn from the family”	1, 2, 3, 7, 10, 12, 14, 15, 16, 18, 21, 23, 24, 25, 26, 34, 35, 37
Emotional complaints: freezing	State of immobility of (part of) the body	13.0%	“Her voice freezes, so she can’t reply” “He is unable to move his feet when he is anxious”	3, 7, 14, 16, 21, 25
Emotional complaints: avoidance	Leaving or refusing certain situations	33.3%	“He was shaking and avoiding eye contact” “Avoiding to initiate social interaction with unfamiliar people” “If guests arrived, he would avoid their presence” “She avoided almost all situations away from home”	1, 2, 4, 5, 6, 8, 12, 14, 15, 16, 20, 21, 22, 24, 25, 28
Emotional complaints: negative affect	Emotional state characterized by negative feelings about oneself or the environment	16.7%	“The rumination about suicide, without the tendency, started” “He confirmed a slightly depressed mood” “Her affect tends to be blunted”	1, 2, 3, 6, 22, 24, 34
Emotional complaints: withdrawn	Behavior that includes social withdrawal and isolation	25.9%	“When other children initiated play, she withdrew” “When she had to change schools during the school period, she became more and more withdrawn”	1, 2, 6, 8, 11, 15, 16, 21, 22, 23, 24, 27, 31, 34
Temperament: behavioral inhibition	Heightened tendency to react with fear, caution, or withdrawal in response to novel stimuli or unfamiliar situations	24.1%	“Shyness for as long as they can remember” “He was shy since early childhood” “From an early age, she appeared quiet and withdrawn”	1, 4, 5, 6, 10, 15, 16, 25, 26, 34, 37, 38
Behavioral complaints	Behaviors that are disruptive, inappropriate, or concerning given the child's age and developmental stage	24.1%	“The twins were hitting each other a lot” “He would never follow his parents instructions” “He had been caught stealing at school”	6, 7, 10, 14, 16, 20, 24, 25, 28, 34
Family history of complaints: extreme shyness	Reported incidence of (symptoms of) selective mutism or extreme shyness within the family	18.5%	“Both parents had been timid and little outspoken as children” “Father described himself as shy by nature” “Mother experienced elements of elective mutism”	9, 10, 22, 23, 25, 27, 32, 35, 37
Family history of complaints: other psychopathology	Reported incidence of other (symptoms of) psychopathology than selective mutism in the family	25.9%	“The patient’s father was treated because of depression” “Father was addicted to alcohol and did not take treatment” “History of anxiety on both parents’ sides of their families”	1, 6, 14, 16, 17, 20, 22, 25, 31, 34, 36
Migration background	Personal or familial history of relocation from a country or region	13.0%	“His family had immigrated to Canada from Iraq” “The family lived abroad until the boy’s fourth year”	8, 13, 14, 24, 29, 30, 33
Bilingualism	When one learns and/or uses two languages at the same time	11.1%	“Gisela spoke both Spanish and English” “Spanish and English were used at home”	1, 8, 12, 21, 30, 33
Traumatic event(s)	Experience that is profoundly distressing or disturbing, often involving a threat to life, bodily or psychological well-being	27.8%	“6 years ago, her father died of a heart attack” “He experienced emotional abuse by his partner” “H was involved in a car accident during a class field trip” “A’s brother was hospitalized for 3 months post-accident”	2, 14, 16, 18, 20, 25, 26, 29, 31, 33, 34, 35, 36
Setting of selective mutism: at school	Absence of speech to teacher(s) and/or peers or friends at school	68.5%	“He did not converse with his classmates” “She was unable to converse with any of her peers” “He would shut down when returning to school after breaks” “The patient ceased to communicate in school” “She failed to speak to anyone while she was in school” “She had never spoken a word in school” “He refused to speak in school”	1, 2, 3, 4, 5, 6, 7, 9, 10, 13, 15, 16, 17, 18, 19, 20, 21, 22, 24, 25, 26, 27, 28, 29, 30, 31, 32, 33, 35, 36, 37, 38
Setting of selective mutism: extended or non-family members	Absence of speech to extended or non-family members in- or outside home	48.1%	“N refused to speak to extended- and non-family members” “She was unable to speak with her physician or dentist” “H would be unable to speak with visitors at home” “Inability to speak in social situations outside of the house” “LM would refuse to interact with neighboring children” “She would not speak in the presence of strangers”	2, 4, 5, 6, 7, 9, 13, 15, 16, 17, 20, 22, 24, 25, 26, 27, 30, 31, 32, 33, 34, 36, 37, 38
Setting of selective mutism: at home	Absence of speech to (one of the) parents or siblings at home	18.5%	“E did not speak to his parents” “He only spoke to his mother, not to his father or siblings”	2, 7,23, 26, 31, 33, 34, 35

* = for all reported frequencies it is possible that more participants were actually experiencing such complaints, but that these complaints were simply not explored or reported by the research teams of the included studies

Regarding the etiology of SM, a positive family history of SM or extreme shyness was reported in ten participants (18.5%). In addition, parents or siblings of fourteen participants were reported to show (symptoms of) psychopathology other than SM (25.9%), like anxiety or depressive disorders. Traumatic life events like hospitalization or death of a close family member or pet, were reported in about a quarter of the participants (*n* = 15, 27.8%). However, only in eight participants these traumatic life events happened before the onset of SM [2, 16, 18, 20, 31, 34, 36]. Last, for two-third of the participants, SM was reported to be present in the school setting (*n* = 37, 68.5%), while for almost half of the participants SM was reported to be present with extended or non-family members in- or outside home (*n* = 26, 48.1%), and for ten participants SM was reported to be present with one or more core family members at home (18.5%).

### Treatments

#### Previous treatment

Previous treatment efforts were reported in 29 participants and were (cognitive) behavioral or psychodynamic in nature and/or entailed adjustments in or outside the classroom with the help of special education services or a speech/language therapist. However, most of these previous treatment efforts were reported to be ineffective. Only the use of an selective serotonin reuptake inhibitor (SSRI) was reported to be a little effective in reducing SM symptoms before current treatment efforts [27].

#### Current treatment

Treatment duration was reported for 30 participants and ranged from one month to two years or 5 to 75 sessions, see Table 4.

**Table 4:** Treatment duration (for n of participants)

	*M (SD)*	*Median*	*Min*	*Max*
Months (*n* = 19)	8.42 (8.07)	4	1	24
Sessions (*n* = *11*)	35.27 (25.53)	25	5	75

#### Psychological interventions

The use of cognitive behavioral treatment (CBT) elements to treat SM was reported in 22 participants (40.7%). At the start of treatment, psycho-education was provided in ten cases to explain the rationale for treatment and to build rapport with the participants and their parents and/or teachers [5–7, 10, 12, 13, 16, 24, 25]. In addition, cognitive restructuring was used in four cases to identify and challenge distorted or irrational thoughts and beliefs, for example that the world, and speaking in it, is unsafe [12, 16, 25, 27]. Moreover, relaxation exercises like diaphragmatic breathing and progressive muscle relaxation to reduce arousal were used in three participants [2, 16, 25], while exposure exercises to feared situations to decrease anxiety and avoidance behaviors and increase speech behavior were conducted in fifteen participants [2, 4, 5, 7, 12, 15, 16, 18, 20, 21, 24, 25, 28, 30]. Also, social skills training to develop and enhance interpersonal skills and improve interaction with others was provided to nine participants [6, 13, 16, 19, 24, 25, 27, 28, 30]. Last, parent training to equip parents with the knowledge, skills, and strategies needed to effectively manage and support their child with SM was provided to the parents of five participants [4, 5, 14, 16, 24]. Contingency management to systematically reinforce desired speech behavior was incorporated into CBT for thirteen participants [4–6, 12, 15, 16, 19–22, 25, 27, 28] and offered as a standalone treatment (behavior therapy (BT)) in five participants [17, 33, 34, 36, 37].

Other psychological interventions reported were creative/art therapy to enhance self-expression in three participants (5.6%) [13, 14, 26], psychodynamic/play therapy to help participants express thoughts and feelings and understand their experiences in six participants (11.1%) [13, 14, 25, 27, 33, 36], and system/family therapy to address the relational dynamics and interactions within the participants’ family in nine participants (16.7%) [6, 7, 10, 23, 25, 27, 29, 34].

#### Pharmacological interventions

Pharmacotherapy was reported to be used to treat SM in 16 participants (29.6%). In all cases but two, an SSRI was prescribed to decrease anxiety symptoms and to increase speech behavior. In one case the SSRI was prescribed to improve depressive symptoms, and in this case the medication did have a positive effect on the individuals’ mood but not on the individuals’ SM [6]. In one case a tricyclic antidepressant was prescribed, with some improvements in mood and social interactions reported, although this participant was still unable to speak at school and with peers [32].

#### Primary treatment outcomes

In total, current treatment effects were described for two-thirds of the participants (*n* = 37; 68.5%). Of them, a total of 25 participants was reported to be SM diagnosis free post-treatment (67.6%), whereas nine participants were reported to still suffer from SM post-treatment (24.3%). For three participants the outcome of the described treatment efforts was not reported (8.1%).

**Fig. 2:**
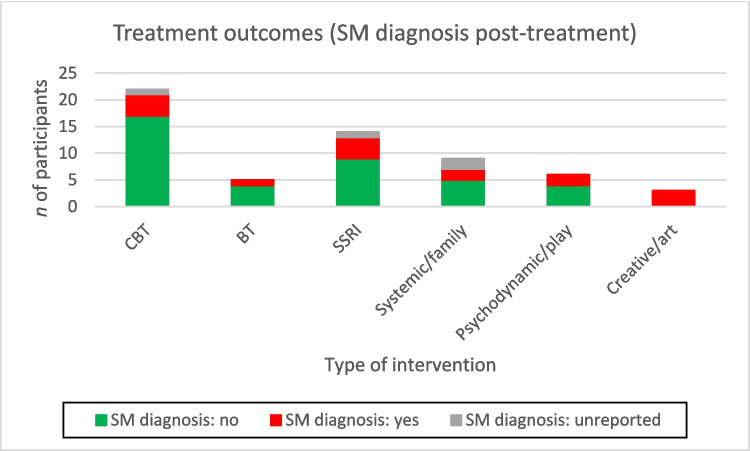
Treatment outcomes for the reported interventions

In Fig. 2, treatment outcomes per specific treatment are shown. Some additional information is worth mentioning. First, out of the 17 participants who were reported to be SM diagnosis free after CBT treatment, the effect of only nine could directly be prescribed to the effects of CBT alone [2, 6, 15, 20, 21, 24, 28, 30]. In all other participants treated with CBT, it remained unclear whether treatment success was due to CBT or the combination with other interventions like pharmacotherapy [4, 5, 10, 12, 16, 27] or psychodynamic/play and/or systemic/family therapy [10, 25, 27]. Second, for two out of four participants who were reported to be SM diagnosis free after contingency management (behavior therapy), the treatment success could also be due to the use of play therapy [33, 36]. Third, in only one of the five success reports of systemic/family therapy this success could solely be prescribed to the systemic intervention [23]. Lastly, in only two cases, the use of medication was reported to be effective as a standalone treatment [26, 31]. As mentioned above, in some participants it remained unclear whether the treatment outcome could directly be prescribed to the effects of pharmacotherapy or the combination with CBT [4, 5, 10, 12, 16, 27].

#### Secondary treatment outcomes

Besides the presence of a SM diagnosis post-treatment, some articles reported on secondary treatment outcomes. For example, improved school attendance was reported in six participants [6, 10, 24, 28, 38], and four participants were reported to be able to move out to college after graduating high school [15, 26, 30, 35], while two participants were reported to have become employed after graduation [14, 38]. Improved independence was reported for five participants, who were reported to be able to do errands, get their driving license, or become a babysitter post-treatment [10, 16, 27, 35]. Although not all participants were SM diagnosis free after treatment, 18 participants were reported to be less socially anxious and less avoidant of social interaction with their family, peers and/or teachers [2, 6, 10, 12, 15, 17, 18, 20, 24–26, 28, 30–33, 35]. However, among the cases who no longer met the criteria for SM post-treatment, three were reported to still show signs of social anxiety [6, 16, 23].

#### Treatment challenges

Different challenges in the treatment process were reported. First and foremost, the chronicity of SM and thus the extensive negative reinforcement history of the selectively mute behavior presented a challenge when designing a treatment program [6, 15, 16, 21, 24, 31]. This long history of secondary gains (i.e., being able to avoid situations like answering questions in class) made it challenging to motivate adolescents to increasingly engage in social interactions. In line with this, low adherence to the treatment or the homework assignments was reported in eleven cases [6, 7, 16, 21, 24, 26, 31, 32, 32, 36, 38]. Strategies like reminders, problem-solving, and goal-setting were generally used to overcome these challenges. Another important challenge to successful treatment was the generalization of what was learned in treatment to other settings [4, 15, 16, 19, 22, 24, 37], often due to lack of opportunities for socialization because of social skills deficits and/or limited peer relationships [10, 15, 16, 19, 24], or due to lack of effective cooperation from school [4, 7, 10, 21, 24]. Social skills training and treatment sessions at school were offered to overcome these challenges in some cases.

### Perspectives on selective mutism

#### Perspectives on causes

From a parent perspective, SM was reported to be reflecting either oppositional behavior or anxiety about school attendance [14], or reflecting nothing else than “being quiet and slow in school” [33]. In one case, parents described SM as a result of being scolded at for speaking during class [13], and in other cases, parents described SM as a way for their child to deal with a traumatic life event [3, 10, 16]. From a teacher's perspective, SM was reported to be due to shyness [8], stubbornness [7, 8, 28], hormonal changes [8], or due to the adolescent deciding not to speak in school yet [20, 37]. A few articles reported on adolescents’ own perspectives on their SM and noted that for them, SM was something that was happening because in some (social) situations they freeze and “their head won’t let them talk” [11, 15, 16]. Other adolescents reported having stopped talking as way to cope with being discriminated or bullied [2, 10], or with other traumatic life events [36]. Some adolescents reported to only talk to people they trust [34] and/or have the feeling that no one cared if they talked [34]. Two adolescents were reluctant to talk because they disliked their voice and did not want others to hear it [2, 32], in one of them this seemed linked to their gender dysphoria [2].

#### Perspectives on maintenance

The function of SM was seen as avoidance or escape from social interaction due to fear of failure or social anxiety by parents [3], therapists/researchers [4, 12, 14–16, 20, 21, 24, 26, 28, 38] and adolescents themselves [6, 16, 27]. In many cases, SM was reported to be maintained by parents [3, 4, 10, 11, 14–16, 18, 20–22, 24, 27, 28, 37] or teachers [3, 4, 8, 10, 11, 15, 17, 20, 22, 26, 30, 32, 33], as they were providing accommodations for the adolescents in or outside home or at school. In addition, peers speaking for the adolescents was reported in some cases [8, 10, 24, 32, 33, 37]. Some adolescents described that over time, their mutism became a habit that was very hard to break [14, 20, 27, 36]. Other described SM as a social role because they were always seen as “the person who does not talk” [5, 15, 20]. In twins, the silence of one seemed to reinforce the silence of the other, as both parties expected the other to start talking first [10].

#### Perspectives on consequences

In school, SM was reported to cause loneliness in adolescents because of the (for them) fear-inducing nature of social interactions [1–3, 10, 13, 20, 28]. Although most adolescents were reported to be accepted by their peers [8, 16, 30, 37], some adolescents were reported to be bullied because of their SM [3, 10, 15]. Some were reported to be at risk of being excluded from school [7, 13, 28]. Others felt ignored by their teachers [10, 20], or very tired and frustrated after a day of school in which they did not speak, eat, drink, or go to the toilet, as they were unable to express their primary needs due to their mutism [10, 13]. One young adult reported worrying about not being able to do all things ‘normal people’ do, like going to university, get a job and be in a relationship [11], and another reported to be denied access to college because she was unable to speak at the interview [26].

At home, SM was reported to be a source of frustration as some adolescents only spoke to some family members (i.e., mother), which was difficult to understand for the other family members (i.e., father or siblings) [7]. In addition, parents perceived their children to be very dependent on them due to their mutism [3, 16, 20], sometimes leading to an almost symbiotic tie between adolescent and parent(s) [14].

#### Perspectives on recovery

For a few adolescents their specific requests for help were reported, like the desire to become less anxious and angry [14], and to be able to talk to everyone [7, 30, 36, 37]. Others reported the desire to make friends [10, 16] or to continue education [31]. In line with this, some parents expressed a desire to learn how to help their child talk to others [10, 16, 24]. Regarding recovery, some adolescents reported on what did not help them, like feeling pushed or pressured into speaking [3, 10, 27]. Instead, they found treatment more helpful when they felt no obligations to respond [3, 10]. Some adolescents reported that it helped them when their peers talked on their behalf [10] or when they could talk to others through online communication [11], as it made them feel like they were accepted for who they are. One adolescent described how the determination of the therapist helped to start talking, as this gave them the feeling that the therapist really cared about them [36]. Looking back, parents reported that at first, they expected their child to grow out of their mutism [5, 8, 16]. When they realized this was not the case, they reported to experience a lot of barriers to specialized care for their children [3]. Once recovered, some adolescents looked back and mentioned they still don’t understand why they didn’t talk before [37].

## Discussion

The current study set out to offer a comprehensive initial overview of the descriptive literature on adolescents and young adults with SM, to provide a better understanding of the characteristics of SM and its treatment in this so far overlooked group of individuals.

### Reflection on results

The reviewed literature was published between 1965 and 2025 and written primarily from a therapist perspective and a cognitive-behavioral approach. The included studies contained 54 unique cases in their adolescence or young adulthood, with two-third aged up to 15 years.

#### Characteristics of SM

Together, the included studies reveal that SM presents differently in adolescents and young adults than in children. First, while SM is more common in girls during childhood [13], the gender distribution was equal in the current group of adolescents and young adults. Second, whereas symptoms in children typically emerge between ages two and four [72], participants in the current study generally showed a later onset of symptoms around age six. Third, the interval between symptom onset and diagnosis was considerably longer in the current group compared to children [13, 27]. This delay may be linked to the relatively high proportion of participants with additional diagnoses, such as social anxiety disorder (SAD) or autism spectrum disorder (ASD), which could have masked SM symptoms. However, the prevalence of these co-occurring conditions was still lower than what is typically reported in children [13, 22, 37, 62]. Fourth, in line with what is found in children, school and interactions with unfamiliar people were common contexts in which speech was absent. Notably, a considerable portion of the adolescents and young adults also experienced mutism with close family members at home and/or reported a family history of SM. Both factors have been linked to less favorable long-term outcomes for SM in previous research [34, 53]. Last, pre- or perinatal risk factors, migration background, and bilingualism were less frequently reported than in childhood cases of SM. Traumatic life events were reported relatively often but typically occurred after symptom onset. Together, these findings suggest that these factors might play a smaller role in SM in adolescence or young adulthood.

#### Treatment of SM

Following from the finding that SM presents differently in adolescents and young adults than in children, the reviewed studies reveal that treatment should be adjusted accordingly. Like for children, cognitive behavioral therapy (CBT) was the most common and effective approach, often used alongside other interventions such as medication or family therapy. In previous literature, CBT is recommended as first-choice treatment for adolescents and young adults with SM, especially because there appears to be a reduction of the impact of early temperament on SM in these age groups specifically [56], while classical and operant conditioning seem to take on an increasing role in SM with age and social experience [28]. In line with this, the reviewed studies showed that CBT included not only exposure tasks and speaking rewards, but also cognitive strategies, relaxation techniques, and social skills training, to address the long standing habit of not speaking and social skill deficits that can follow from it. Two-thirds of the current sample no longer met the criteria for SM after treatment (68.5%), slightly less than what was found in a recent systematic review among children aged 3–14 years (78% [34]). This indicates that SM might be more therapy-resistant in adolescents and young adults. However, while some symptoms like shyness persisted, improvements were often noted in independence, school attendance, and social anxiety.

#### Perspectives on SM

Given that SM manifests differently in adolescents and young adults than in children, the reviewed studies highlight the need to assess their lived experiences to better understand their specific (treatment) needs. Parents, teachers, therapists, researchers, and also adolescents and young adults themselves often viewed SM as a way to avoid anxiety-provoking social situations, although some adolescents and young adults in this study also linked it to freezing, coping with bullying or trauma, or discomfort with their voice, suggesting age-related differences in causes compared to children. SM was often sustained by accommodations from others and sometimes became a social role. Experiences at school varied, with some feeling accepted and others facing loneliness, exclusion or bullying. Some felt their SM hindered their social and academic growth and caused frustration over not being able to express needs. Many wanted to overcome anxiety, build friendships, and speak freely. At home, mutism was tied to dependence on parents, and while families initially hoped it would resolve on its own, they often sought specialist help when it persisted. Importantly, treatment was perceived as more effective when there was no pressure to speak.

### Implications for clinical practice

Due to growing diagnostic awareness of SM and the improved understanding of its possible co-occurrence with other conditions such as ASD, it is expected that adolescents and young adults with SM will increasingly present in clinical practice. This poses challenges for clinicians who might be used to treating SM in younger children, as adolescents and young adults have a longer history of negative reinforcement of non-speaking behavior and avoidance of social interaction, and are therefore in general more difficult to treat [34, 46]. This is also reflected by the long treatment durations found in the current study (up to two years or 75 sessions), or as one school psychologist mentioned “the longer the prevalence of SM, the longer the duration of treatment” [40]. Fortunately, the reviewed literature provides some suggestions for the assessment and treatment of SM in adolescents and young adults specifically. First of all, the long lag time between age of onset and age of diagnosis of 40 months in the current sample confirms that SM is sometimes overlooked among adolescents and young adults. Therefore, a first step towards better care for this age group specifically, is for clinicians to be aware of SM among all ages, so treatment can be offered before SM becomes habitual or a social role and consequently even harder to treat.

Regarding assessment and treatment of SM, it is recommended to assess *if* SM is present, using standardized tools like the Selective Mutism Questionnaire (SMQ; [8, 54]) and the Structured Clinical Interview DSM-5 – Junior (SCID-5-Junior; [73]). The finding that almost half of the current sample had additional diagnoses, such as SAD or ASD, suggests that in some cases SM may be associated not only with social anxiety but also with broader social difficulties [44]. We therefore concur with Muris and Ollendick [44] in their argument that the potential contribution of ASD to SM should be acknowledged by disregarding the ambiguous fifth DSM-5 criterion stating that the primary feature of SM—failure to speak—should not occur exclusively in the context of ASD, thereby allowing SM and ASD to be classified as co-occurring conditions.

We also endorse the recommendations of Oerbeck and colleagues [47] to assess *why* SM is present, using a case conceptualization. This case conceptualization can be used to determine the focus of treatment, either on the behavioral component of CBT like in preferred treatments for younger children with SM, or on other CBT components where considered appropriate, like cognitive restructuring (focused on maladaptive thoughts about speaking), relaxation exercises (focused on freezing in situations where speech is required), or social skills training (focused on deficits in social contact or relations with others). In all cases, the reviewed literature showed that treatment should be focused on sustaining factors as well, for example by targeting accommodating behaviors of parents, teachers and siblings or peers (e.g., SPACE; [41]). To motivate adolescents and young adults, treatment should be focused on their own treatment goals, that might be more about gaining independence than about speaking per se. This indicates that treatment outcomes should not only be assessed using standardized outcomes like the SMQ, but also by using quality of life or self-efficacy measures like the WHO Quality Of Life checklist [77] or the Self Efficacy Scale for Children (SEC-Q; [43]). The use of digital communication should be considered to aid treatment, given that some adolescents and young adults in the current sample reported that it helped them to stay in touch with and feel accepted by others. However, for some adolescents and young adults with SM, the use of non-verbal communication (written and digital) is in itself anxiety inducing [20], and should therefore be considered a treatment goal to help them get back in touch with the people around them.

### Strengths and limitations

The current study has a few strengths that make it particularly useful in a field where evidence from large-scale studies is sparse due to the rare nature of SM in adolescents and young adults. First, by including studies with detailed descriptions of individual circumstances we were able to capture information that might not become evident in larger quantitative studies, like treatment challenges and lived experiences of adolescents and young adults with SM. Second, by including studies spanning a few decades and different parts of the world, we were able to collect information from different theoretical and cultural perspectives. Still, one-third of the included studies were published in the last ten years and four-fifth included a Western, Educated, Industrialized, Rich, and Democratic (WEIRD) sample. Above that, this study holds a bias towards adolescents and young adults who did not benefit from previous treatments and who have returned to therapy. The study missed those who benefited from treatment immediately and those who did not return after an unsuccessful treatment. Thus, the findings of this study may not be generalizable to the broader population seen in routine clinical practice. Moreover, the diversity in methodologies, reporting styles, and contexts across the included studies made it challenging to synthesize the findings. For example, there was variability in depth and type of information reported per included study, and often it was unclear whether something was not reported because it did not occur, or did occur but was (un)intentionally not reported. Although we tried to take this problem into account by reporting as concise as possible, the numbers found in the current study might still be an underestimation of what is seen in routine clinical care.

### Directions for future research

This systematic review is a consolidation of 60 years of research in adolescents and young adults with SM, and includes descriptive research mainly in the form of case studies or case reports. However, studies differed remarkedly in their reliance on child-, parent-, and/or clinician- based reports across various outcomes, including (remission of) SM diagnosis, symptoms and symptom reduction, and other domains of functioning (e.g., peer relationships). Therefore, future research should add standardized measures of SM to improve comparison of findings across studies. On top of that, future studies adopting the perspective of adolescents and young adults are needed, as such research remains scarce. Gaining further insight into which factors contribute to the persistence of SM, which treatment approaches are most effective in this age group, and which challenges are experienced by adolescents and young adults is essential for improving care and outcomes in this population.

## Conclusions

The present study is the first systematic review of literature describing adolescents and young adults with SM. In summary, SM presents differently in adolescents and young adults than in children. Adolescents and young adults tend to experience a later onset and a longer diagnostic delay, possibly linked to comorbid disorders like SAD and ASD, which could have masked SM symptoms. Compared to children, adolescents and young adults with SM have a longer history of negative reinforcement of their non-speaking behavior, due to experiences such as bullying or social isolation or due to accommodations in the home and/or school context. Consequently, SM in adolescents and young adults seems to be more therapy-resistant and requires therapists to be patient when using an evidence-based approach. In addition, where younger children with SM can still navigate social interaction non-verbally through means of play, verbal communication is an inherent aspect of socialization among adolescents and young adults. These developmental differences underscore the importance of adopting age-appropriate approaches in both research and treatment of SM among older youth.

## Data Availability

No datasets were generated or analysed during the current study.

## Appendix 1

**Table:**

Section and Topic	Item #	Checklist item	Page where item is reported
Title			
Title	1	Identify the report as a systematic review	1
Abstract			
Abstract	2	See the PRISMA 2020 for Abstracts checklist	2
Introduction			
Rationale	3	Describe the rationale for the review in the context of existing knowledge	3–6
Objectives	4	Provide an explicit statement of the objective(s) or question(s) the review addresses	6
Methods			
Eligibility criteria	5	Specify the inclusion and exclusion criteria for the review and how studies were grouped for the syntheses	7–8
Information sources	6	Specify all databases, registers, websites, organisations, reference lists and other sources searched or consulted to identify studies. Specify the date when each source was last searched or consulted	7
Search strategy	7	Present the full search strategies for all databases, registers and websites, including any filters and limits used	7
Selection process	8	Specify the methods used to decide whether a study met the inclusion criteria of the review, including how many reviewers screened each record and each report retrieved, whether they worked independently, and if applicable, details of automation tools used in the process	7–8
Data collection process	9	Specify the methods used to collect data from reports, including how many reviewers collected data from each report, whether they worked independently, any processes for obtaining or confirming data from study investigators, and if applicable, details of automation tools used in the process	8
Data items	10a	List and define all outcomes for which data were sought. Specify whether all results that were compatible with each outcome domain in each study were sought (e.g. for all measures, time points, analyses), and if not, the methods used to decide which results to collect	8, 12–14
	10b	List and define all other variables for which data were sought (e.g. participant and intervention characteristics, funding sources). Describe any assumptions made about any missing or unclear information	8, 12–14
Study risk of bias assessment	11	Specify the methods used to assess risk of bias in the included studies, including details of the tool(s) used, how many reviewers assessed each study and whether they worked independently, and if applicable, details of automation tools used in the process	8, 9, 10, 11
Effect measures	12	Specify for each outcome the effect measure(s) (e.g. risk ratio, mean difference) used in the synthesis or presentation of results	n/a
Synthesis methods	13a	Describe the processes used to decide which studies were eligible for each synthesis (e.g. tabulating the study intervention characteristics and comparing against the planned groups for each synthesis (item #5))	10
	13b	Describe any methods required to prepare the data for presentation or synthesis, such as handling of missing summary statistics, or data conversions	10
	13c	Describe any methods used to tabulate or visually display results of individual studies and syntheses	10
	13d	Describe any methods used to synthesize results and provide a rationale for the choice(s). If meta-analysis was performed, describe the model(s), method(s) to identify the presence and extent of statistical heterogeneity, and software package(s) used	10
	13e	Describe any methods used to explore possible causes of heterogeneity among study results (e.g. subgroup analysis, meta-regression)	10
	13f	Describe any sensitivity analyses conducted to assess robustness of the synthesized results	n/a
Reporting bias assessment	14	Describe any methods used to assess risk of bias due to missing results in a synthesis (arising from reporting biases)	n/a
Certainty assessment	15	Describe any methods used to assess certainty (or confidence) in the body of evidence for an outcome	n/a
Results			
Study selection	16a	Describe the results of the search and selection process, from the number of records identified in the search to the number of studies included in the review, ideally using a flow diagram	8
	16b	Cite studies that might appear to meet the inclusion criteria, but which were excluded, and explain why they were excluded	8
Study characteristics	17	Cite each included study and present its characteristics	12–14
Risk of bias in studies	18	Present assessments of risk of bias for each included study	n/a
Results of individual studies	19	For all outcomes, present, for each study: (a) summary statistics for each group (where appropriate) and (b) an effect estimate and its precision (e.g. confidence/credible interval), ideally using structured tables or plots	12–26
Results of syntheses	20a	For each synthesis, briefly summarise the characteristics and risk of bias among contributing studies	n/a
	20b	Present results of all statistical syntheses conducted. If meta-analysis was done, present for each the summary estimate and its precision (e.g. confidence/credible interval) and measures of statistical heterogeneity. If comparing groups, describe the direction of the effect	n/a
	20c	Present results of all investigations of possible causes of heterogeneity among study results	n/a
	20d	Present results of all sensitivity analyses conducted to assess the robustness of the synthesized results	n/a
Reporting biases	21	Present assessments of risk of bias due to missing results (arising from reporting biases) for each synthesis assessed	n/a
Certainty of evidence	22	Present assessments of certainty (or confidence) in the body of evidence for each outcome assessed	n/a
Discussion			
Discussion	23a	Provide a general interpretation of the results in the context of other evidence	27–29
	23b	Discuss any limitations of the evidence included in the review	30–31
	23c	Discuss any limitations of the review processes used	30–31
	23d	Discuss implications of the results for practice, policy, and future research	29–32
Other information			
Registration and protocol	24a	Provide registration information for the review, including register name and registration number, or state that the review was not registered	n/a
	24b	Indicate where the review protocol can be accessed, or state that a protocol was not prepared	n/a
	24c	Describe and explain any amendments to information provided at registration or in the protocol	n/a
Support	25	Describe sources of financial or non-financial support for the review, and the role of the funders or sponsors in the review	1
Competing interests	26	Declare any competing interests of review authors	1
Availability of data, code and other materials	27	Report which of the following are publicly available and where they can be found: template data collection forms; data extracted from included studies; data used for all analyses; analytic code; any other materials used in the review	1
